# Controlled fabrication of nanoscale wrinkle structure by fluorocarbon plasma for highly transparent triboelectric nanogenerator

**DOI:** 10.1038/micronano.2016.74

**Published:** 2017-01-30

**Authors:** Xiaoliang Cheng, Liming Miao, Zongming Su, Haotian Chen, Yu Song, Xuexian Chen, Haixia Zhang

**Affiliations:** 1 National Key Laboratory of Nano/Micro Fabrication Technology, Institute of Microelectronics, Peking University, Beijing 100871, China

**Keywords:** fluorocarbon plasma, nanostructure fabrication, triboelectric nanogenerator, wrinkle structure

## Abstract

In this paper, we report a novel nanoscale wrinkle-structure fabrication process using fluorocarbon plasma on poly(dimethylsiloxane) (PDMS) and Solaris membranes. Wrinkles with wavelengths of hundreds of nanometers were obtained on these two materials, showing that the fabrication process was universally applicable. By varying the plasma-treating time, the wavelength of the wrinkle structure could be controlled. Highly transparent membranes with wrinkle patterns were obtained when the plasma-treating time was <125 s. The transmittances of these membranes were >90% in the visible region, making it difficult to distinguish them from a flat membrane. The deposited fluorocarbon polymer also dramatically reduced the surface energy, which allowed us to replicate the wrinkle pattern with high precision onto other membranes without any surfactant coating. The combined advantages of high electron affinity and high transparency enabled the fabricated membrane to improve the performance of a triboelectric nanogenerator. This nanoscale, single-step, and universal wrinkle-pattern fabrication process, with the functionality of high transparency and ultra-low surface energy, shows an attractive potential for future applications in micro- and nanodevices, especially in transparent energy harvesters.

## Introduction

Patterned surface structures on the nano- or micrometer scale have a significant role in the properties of a material, including physical, mechanical, electrical, and optical^1^. The fabrication process for these patterns has been traditionally realized by photolithography, printing processes, embossing, or writing techniques, of which the relatively high cost and low throughput limit their application for producing complex topologies over large areas. Thereby, self-assembled patterns for large-area patterning, which generally employ physical–chemical or mechanical instabilities within a constrained system to form highly ordered structures, have attracted great attention for many years^2–7^. Among these methods, mechanically inducing a wrinkle structure on a bilayer system is especially suited to creating highly ordered microstructures across a large surface and features convenient fabrication and a tunable wavelength by adjusting the thickness of the stiff layer and the pre-strain^8–12^. As a result, wrinkle structures have been used in various applications, such as smart adhesion, liquid/cell shaping, particle assembly, optical surfaces, flexible electronic devices, and energy harvesters^13–20^. To date, most studies involving wrinkling to create ordered structures rely on plasma or ultraviolet-ozonolysis (UVO) oxidation and metal deposition to create the stiff skin. Although these approaches are quite practical, the material properties of the substrate during oxidation can significantly alter the typical morphologies and even block the formation of the wrinkle structure (that is, the process does not have universality to different materials), whereas the deposition of the metal layer would greatly decrease the transparency of the substrate^21,22^. All of these drawbacks limit the practical application of this method in specific occasions that require high transparency and other materials. Alternatively, replica molding (REM) is an efficient and simple method for the duplication of the information (that is, shape, morphology, and structure) present in the surface of a mold^23,24^. The wrinkle-fabrication process can be further simplified and the cost can be reduced by using a fabricated wrinkle mold to duplicate a wrinkle pattern via REM. However, the replication mold needs a low surface energy to avoid adhesion, and the oxygen plasma and UVO oxidation method can increase the surface energy. Surface energy can be decreased by some surfactant coatings; however, these coatings unfortunately will pollute the surface and reduce the precision of the replication. Both reasons make it inconvenient to use the oxygen plasma and UVO method in the fabrication of wrinkle molds.

Energy-harvesting technology that uses the coupling effects of contact-electrification and electrostatic induction, called a triboelectric nanogenerator (TENG), has been considered a promising alternative for renewable and green energy applications, such as self-powered electronic devices, touch sensors, and artificial skins, due to its advantages of high output power, scalability, simple design, and cost-effective fabrication^25–30^. In addition, transparent TENGs using indium tin oxide (ITO) or graphene as electrodes have been achieved, which enable applications that require high transparency such as touch screens^31–35^. Our previous work shows that using C_4_F_8_ to treat uncured PDMS could deposit fluorocarbon polymer and a wrinkle structure in single step^18–20^, which had the advantage of significantly increasing the performance of the flexible TENG. This enhancement was attributed to the roughness of the wrinkle pattern and high electron affinity of the fluorocarbon polymer^36,37^. However, the wrinkle structure was large (that is, a dozen or dozens of micrometers) and disordered, which decreased the transparency of the membrane and restricted its application for applications requiring nanoscale and regular structures. Although using a flat surface could provide the highest transparency, the performance of the TENGs will be weakened to some degree^38^. Notably, previous work reported that a PDMS surface with nanopattern sizes <310 nm could result in high transmittances (above 85%) owing to an effective graded refractive index profile^32,33^. Using a structure of several hundred nanometers provides us with an effective way to realize high-performance and highly transparent TENGs.

We propose a novel wrinkle-structure fabrication process by depositing fluorocarbon plasma on pre-stretching substrates. This method possesses three advantages over previous works. First, using the fluorocarbon plasma to deposit fluorocarbon polymer as the stiff layer is universally applicable and capable of fabricating wrinkle patterns on more materials compared with the oxidation process. The highly desirable property of transparency of the fluorocarbon polymer does not affect the transmittance of the substrate compared with the metal deposition. Second, a wrinkle structure with wavelength of <200 nm is obtained through this process, which makes the transmittance of the patterned membrane in the visible region almost indistinguishable from a flat substrate. Third, the ultra-low energy of the fluorocarbon polymer on the substrate stabilizes the surface, which enables the patterned substrate to serve as a mold to efficiently transfer the wrinkle structure to another membrane without requiring surfactant. Due to these features, the processed membranes are assembled to form TENGs, and a significant improvement is achieved compared with the flat membrane, without reducing the transmittance of the whole device.

## Materials and methods

### Sample preparation

**Figure 1:**
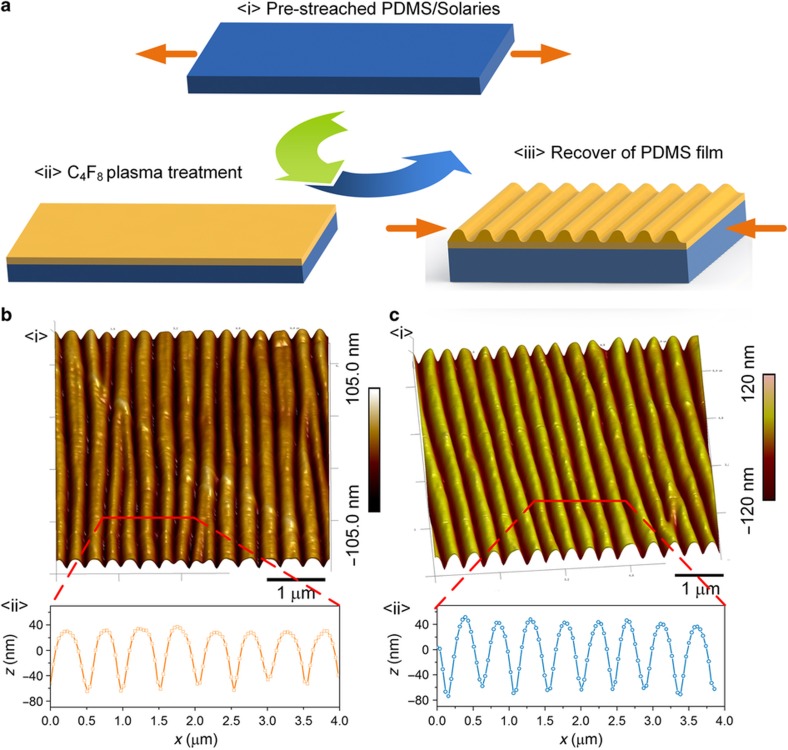
(**a**) Schematic diagram of the fabrication process for the proposed nanoscale wrinkle structure by C_4_F_8_ plasma treatment. (**b**) The AFM image and the sectional profile of the fabricated wrinkle structure on the PDMS substrate with a plasma-treating time of 250 s. (**c**) The AFM image and the sectional profile of the fabricated wrinkle structure on the Solaris membrane with a 250 s plasma-treating time. AFM, atomic force microscopy; PDMS, poly(dimethylsiloxane).

The elastomer and cross-linker of PDMS (Dow Corning, Midland, MI, USA, Sylgard 184) and Solaris (Smooth-on, Macungie, Pennsylvania, PA, USA) were each thoroughly mixed in a 10:1 ratio (*w/w*) and degassed for 30 min to remove the interface bubbles. Subsequently, the samples were heated at a high temperature of 70 °C for 1 h to cure the liquid PDMS and Solaris into solid PDMS and Solaris membranes, respectively. The thickness of these two types membranes were both controlled at ~1 mm. The fabrication diagram for the wrinkle structure is shown in Figure 1a. Rectangular samples of approximately 2×4 cm were cut with a surgical blade. Uniaxial strain was then applied by clamping either end and stretching the membrane along its length as shown in Figure 1a, <i>. In our experiments, the pre-strain was fixed at 10% for all samples. The pre-stretching sample was placed in the center of ICP equipment (ICP-2B, Beijing Jinshengweina Technology Co., Ltd, Beijing, China) etcher for fluorocarbon plasma treatment using C_4_F_8_ (Figure 1a, <ii**>**). The platen power and coil power were controlled to be 0 and 200 W, respectively. The pressure was set at 3 Pa and, the inlet flow rate was fixed at 40 sccm, although the plasma-treating time was varied from the 30 to 500 s to change the thickness of the fluorocarbon polymer. Finally, the sample was removed from the oven, and the strain was carefully released to form the regular wrinkle structure, as illustrated in Figure 1a, <iii**>**.

### Transferring the wrinkle to another substrate

**Figure 2:**
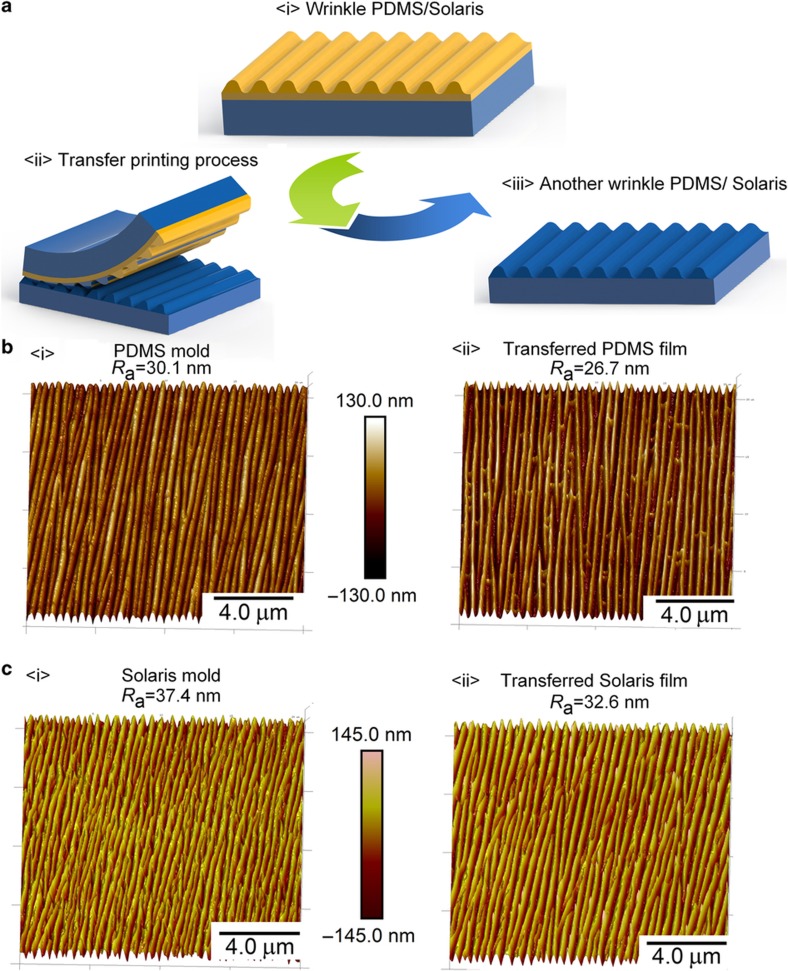
(**a**) The fabrication diagram for using the fabricated wrinkle membrane to replicate its structure to another membrane by transfer printing without using surfactant. (**b**) The AFM images of the PDMS mold and the transferred PDMS film by this mold. (**c**) The AFM images of the Solaris mold and the transferred Solaris film from this mold.

First, we cast the prepared liquid PDMS and Solaris in section ‘Fabrication of wrinkle structures on different substrates’ onto two glass slides and spread them uniformly. Next, the fabricated membranes were placed on the liquid PDMS and Solaris (Figure 2a). The two samples were heated at the high temperature of 70 °C for 1 h to obtain solid PDMS and Solaris membranes. After thermal cross-linking, the PDMS and Solaris films were simply peeled from the substrate.

### Measurement and analysis

The processed wrinkle structure was characterized by an atomic force microscope (Dimension ICON, Bruker Corp., Karlsruhe, Germany) in PeakForce mode, which is a featured mode of Bruker Corp. that could provide the highest resolution. Then, the results were analyzed by Nanoscope Analysis 1.5 software (Bruker Corp., Karlsruhe, Germany). The transmittance of the samples was measured using UV–vis spectroscopy with a UV-3600 spectrophotometer (Shimadzu, Kyoto, Japan). The output voltage of the TENG was measured via a digital oscilloscope (Agilent DSO-X 2014A, Agilent Technologies Inc., Santa Clara, CA, USA) using a 100 MΩ probe (HP9258, Prokit's Industries Co., Ltd., New Taipei City, Taiwan, China), and the current was amplified by a SR570 low-noise current amplifier (Stanford Research Systems, Inc., Sunnyvale, CA, USA). The upper surface of the TENG was fixed. Then, a sinusoidal signal with an amplitude of 1.5 V was generated from the signal-source module of the oscilloscope and amplified by an amplifier (YE5871A, SINOCERA, Shanghai, China) to power the modal shaker (JZK-10, SINOCERA, Shanghai, China), which provided a periodic and stable external force to the device.

## Results and discussion

### Fabrication of wrinkle structures on different substrates

The fabrication process for the regular nanoscale wrinkle pattern on different substrates is diagramed in Figure 1. Two representative elastic materials (PDMS and Solaris) were selected to show the universality of our method. Uniaxial strain was applied by clamping either end and stretching the membrane along its length, as shown in Figure 1a, <i>. In our experiments, the pre-strain was fixed at 10% for all samples. The prestretched sample was placed in the center of an inductively coupled plasma (ICP) etcher for fluorocarbon plasma treatment using C_4_F_8_ (Figure 1a, <ii>). The sample was removed from the oven and the strain was carefully released to form the regular wrinkle structure as illustrated in Figure 1a, <iii>.

Because of instability of the bilayer system, the typical wrinkle structures caused by uniaxial stress are sinusoidal grooves. Figure 1b, <i> shows the AFM image of wrinkle topologies on the PDMS substrate with a plasma-treating time of 250 s. From the sectional profile, we could find that the average wavelength of this sample was ~0.54 μm (Figure 1b, <ii>). Similarly, Figure 1b presents the AFM image of wrinkle topologies on the Solaris substrate with the same treating time. The average wavelength for this sample was ~0.49 μm. Compared with the PDMS substrate, the wavelength of the wrinkle structure on Solaris was smaller. Meanwhile, we noticed that the wrinkle profile on Solaris was closer to a sinusoidal shape. In the region of low deformation, assuming a homogeneous film and bulk materials with perfect adhesion at the interface, *λ* can be described by^8^:

(1)λ=2πhf(Ef¯3Es¯)1/3

where $\bar{E}$ is the plane strain modulus given by $\bar{E}=\frac{E}{1-\nu^{2}},$ *E* is the Young’s modulus, *ν* is the Poisson ratio, *h*_f_ represents the stiff film thickness. The subscripts f and s denote the stiff film and the soft substrate, respectively. In this experiment, the thickness of the substrate *h*_s_≫*h*_f_. *ε* is the strain applied to the bilayer, and the critical strain is defined by:

(2)εc=−14(3Es¯Ef)2/3

Equation (1) indicates that the pattern periodicity is inversely proportional to the Young’s modulus of the substrate. Since the wavelength of Solaris was smaller than that of the PDMS, it can be concluded that the Young’s modulus of Solaris was larger than that of the PDMS. This conclusion can be validated by the test results of PDMS and Solaris using the stress–strain curves shown in Supplementary Figure S1. The Young’s moduli for Solaris and PDMS were calculated to be 1.08 and 1.36 Mpa, respectively. In addition, Equation (2) indicates that a minimum strain is needed for wrinkles to appear. The pre-strain adopted in this work serves as the compressive strain once released.

### A controllable nanoscale wrinkle pattern

**Figure 3:**
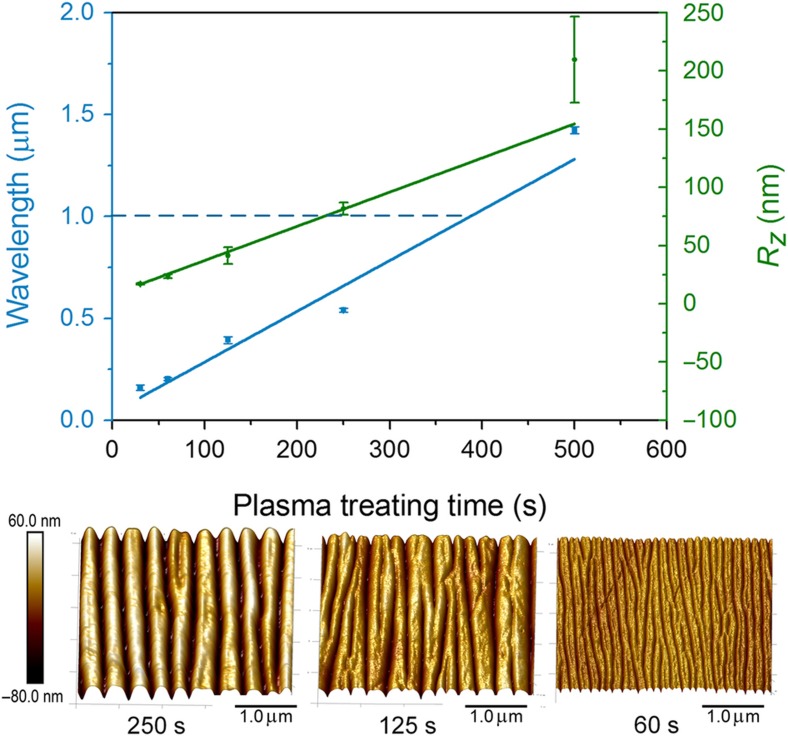
The effect of plasma-treating time on the wrinkle topology for the PDMS membranes: the wavelength, *λ*, and the surface roughness, *R*_z_, as a function of the plasma-treating time; the selected AFM images of wrinkle structure with a plasma-treating time of 250, 125, and 60 s.

In addition to the property of the substrate, the *h*_f_ has a proportional effect on the wrinkle wavelength. The deposition velocity of fluorocarbon polymer is a constant without changing the parameter of the ICP. Therefore, the wavelength can be manipulated by controlling the plasma-treating time. The characterization of fabricated wrinkle structures on the PDMS substrate is plotted in Figure 3. As predicted by Equation (1), the wavelength increased linearly with the plasma-treating time, with an *R*^2^ of ~0.97. According to the result of the linear fit, the wavelength would be <1 μm when the plasma-treating time is shorter than 400 s. The smallest wavelength of ~0.16 μm was reached at the time of 30 s. The amplitude of the wrinkle pattern is represented by the roughness (*R*_z_) along a certain profile. *R*_z_ is defined by:

(3)Rz=∑i=15ypi+∑i=15yvi5

where *y*_p*i*_ is the number *i* highest peak along the profile, and *y*_v*i*_ is the number *i* lowest valley along the profile. The *R*_z_ increased linearly with the plasma-treating time. Meanwhile, the AFM images of wrinkle patterns under 250, 120, and 60 s plasma-treating times indicated that the defects on the wrinkle structure obviously decreased when increasing the plasma-treating time. This can be attributed to the insufficient thickness and partial coverage of the fluorocarbon polymer at a shorter treating time, as reported by our previous work^18^.

**Figure 4:**
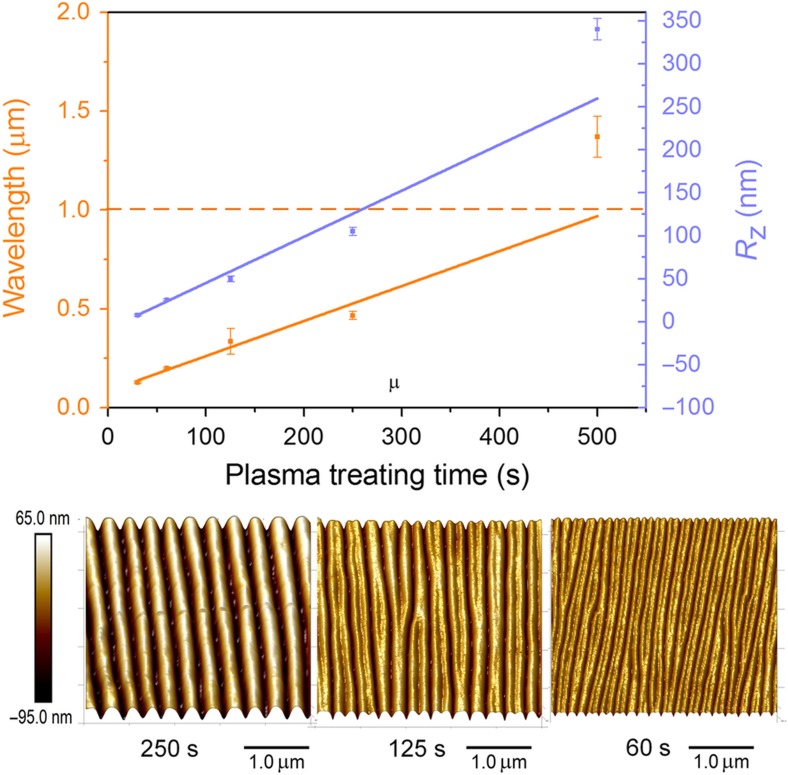
The effect of plasma-treating time on the wrinkle topology for the Solaris substrates: the wavelength, *λ*, and the surface roughness, *R*_z_, as a function of the plasma-treating time; the selected AFM images of wrinkle structure with a plasma-treating time of 250, 125, and 60 s. AFM, atomic force microscopy.

Figure 4 shows the corresponding characterization of fabricated wrinkle structures on the Solaris substrate. Similar to the wrinkle structure on the PDMS substrate, a linear relationship between the wavelengths and plasma-treating times was obtained, with an *R*^2^ of ~0.97. With a larger Young’s modulus, the wavelength of the Solaris substrate under the same plasma-treating time was smaller compared with those of the PDMS substrate. The smallest wavelength, ~0.13 μm, was observed at the 30 s plasma treatment time. The *R*_z_ also increased linearly with the plasma-treating time. In addition, although more defects were observed at a shorter plasma-treating time, as shown in the AFM images in Figure 4, the defects on the Solaris substrates were obviously less than that on the PDMS substrate. The reason for this difference may be that there is better adhesion between the fluorocarbon polymer and Solaris.

### Characterization of transmittance

**Figure 5:**
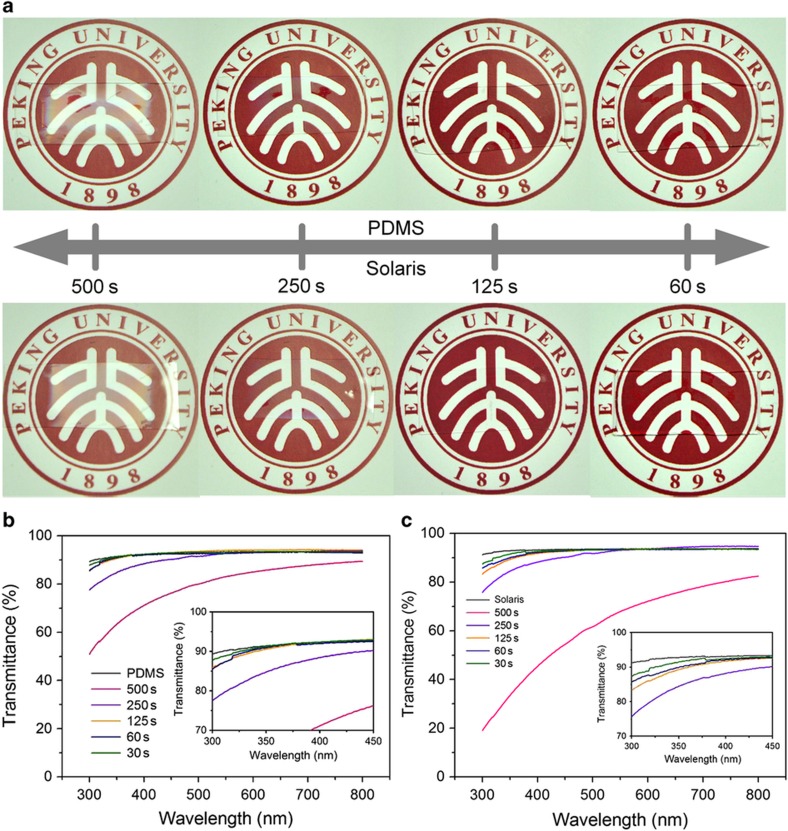
(**a**) The photographs of the wrinkle-patterned PDMS and Solaris membranes with different plasma-treating times to exhibit their high transparency. (**b**) The UV–vis spectrums of the wrinkle-patterned PDMS membranes with different plasma-treating times. (**c**) The UV–vis spectrums of the wrinkle-patterned Solaris samples with different plasma-treating times. UV–vis, ultraviolet–visible.

This wrinkle-structure fabrication method could easily accomplish pattern wavelengths of hundreds of nanometers, which made it possible to provide excellent transmittance to the substrate. Figure 5a presents photographs of the membranes with a wrinkle structure on the PDMS and Solaris substrates under different plasma-treating times. At a plasma-treating time of 500 s, both PDMS and Solaris membranes exhibited iridescent colors on their surfaces, showing strong scattering to the incident light on their wrinkled surfaces. With the decrease of plasma-treating time, the transparency of these membranes had obvious improvement. Although there were still some iridescent colors on the substrates with 250 s of plasma treatment, the iridescent color could not be observed when the duration of plasma treatment lasted for 125 or 60 s. Ultraviolet–visible (UV–vis) spectroscopy was used to further test the transmittance of each sample with different plasma-treating times. As plotted in Figure 5b, the transmittance of PDMS membranes increased markedly when the plasma-treating time decreased from 500 to 250 s, while with a treating time of <125 s, the transmittance became difficult to distinguish from the flat PDMS film. For the Solaris substrates, as shown in Figure 5c, the transmittance of the sample with a 500 s plasma-treating time decreased sharply from 80 to 20% when the wavelength of incident light changed from 800 to 300 nm. Apart from this sample, the other samples showed a similar tendency to the PDMS substrates, and a similar transmittance to the flat Solaris membrane was achieved.

### Serving as a transfer mold

In addition to the high transparency enabled by the nanoscale wrinkle, another meaningful advantage of this method is the ultra-low surface energy of the fluorocarbon polymer on the surface, which makes it possible to use the fabricated wrinkle pattern as a transfer mold. To demonstrate this capability, we used the patterned PDMS/Solaris membrane to fabricate another membrane with inverse structure, as diagramed in Figure 2a (detailed fabrication process is given in Subsection ‘A controllable nanoscale wrinkle pattern’). Figure 2b presents the comparison of the AFM images of the PDMS mold and the corresponding transferred PDMS film. The transferred PDMS film had an opposite structure compared with the mold, although its roughness *R*_a_ dropped slightly from 30.1 to 26.7 nm. The *R*_a_ here can be defined by:

(4)Ra=1N∑j=11|yj|

where *y*_*j*_ is the absolute values of the surface height deviations of point *j* within the image measured from the mean plane and *N* is the total number of points within the image. Figure 2c gives the comparison of the AFM images of the Solaris mold and the corresponding transferred Solaris membrane. The transferred Solaris film also had an inverse structure compared with the Solaris mold, and its roughness decreased slightly from 37.4 to 32.6 nm. The purpose of this demonstration shows that the mold does not rely on the substrate material, which indicates that both PDMS and Solaris membranes could serve as a mold to transfer wrinkle structures to other materials in a single step.

### Highly transparent and high-performance TENG

**Figure 6:**
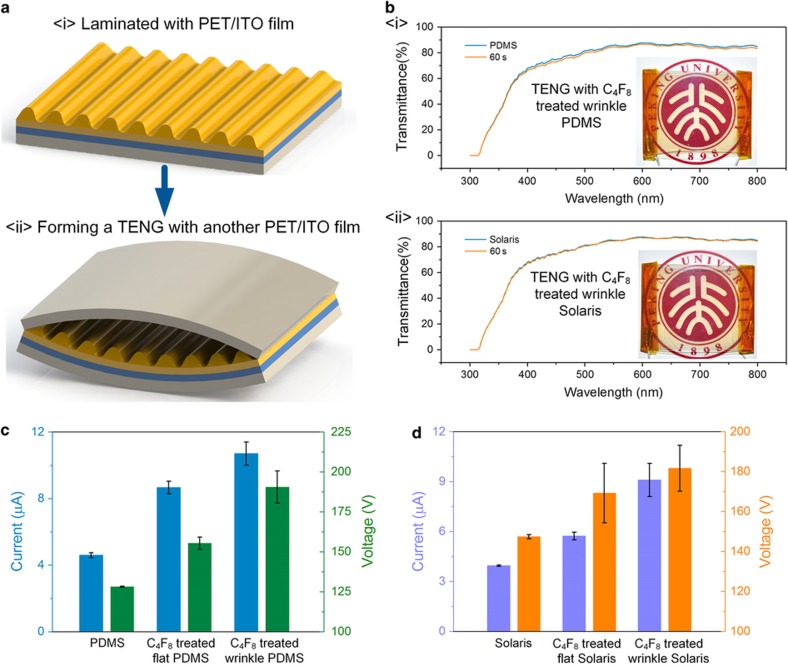
(**a**) The fabrication diagram for using the wrinkle-patterned membrane to assemble a highly transparent and high-performance TENG. (**b**) The comparisons of UV–vis spectrums of PDMS with 60 s plasma-treated wrinkle-pattern PDMS (<i>) and Solaris with 60 s plasma-treated wrinkle-pattern Solaris (<ii>), although all membranes were laminated with a PET/ITO film. (**c**) The comparisons of output voltage and current of TENGs using untreated PDMS, C_4_F_8_-treated flat PDMS, and the C_4_F_8_-treated wrinkle PDMS as the contact-electrification material. (**d**) The comparisons of output voltage and current of TENGs using untreated Solaris, C_4_F_8_-treated flat Solaris, and the C_4_F_8_-treated wrinkle Solaris as the contact-electrification material.

To demonstrate the superiority of this wrinkle pattern in the application of transparent devices, the fabricated PDMS and Solaris membranes were laminated with commercial PET/ITO films and formed into a TENG with another PET/ITO film, as diagramed in Figure 6a. On the basis of the above results, membranes with a 60 s plasma-treating time were employed to obtain high transparency. Figure 6b shows that the prepared samples showed similar transmittances with their corresponding flat materials (above 85% in the range of 540 to 800 nm). The inserted images of the TENGs also show the high transparency of the contact material. In addition, the wrinkle topologies on these samples could be used to improve the performance of the TENG. As illustrated in Figure 6c, compared to the TENG using flat PDMS, the output voltage and current of the TENG using C_4_F_8_ plasma-treated wrinkle PDMS increased from 128 V and 4.6 μA to 191 V and 10.7 μA, an increase of 50 and 132%, respectively. Similarly, the output voltage and current of the TENG using C_4_F_8_ plasma-treated wrinkle Solaris were 181 V and 9.1 μA, which were improved of 24 and 128%, respectively, compared with the TENG using flat Solaris, as shown in Figure 6d. It should be mentioned that both the wrinkle pattern and the fluorocarbon layer contribute to the enhancement of the electric performance of the TENG. This conclusion can be obtained from the electric performance comparison in Figures 6c and d, from which the electric performance of the fluorocarbon deposited TENGs (that is, C_4_F_8_ plasma-treated flat PDMS in Figure 6c and C_4_F_8_ plasma-treated flat Solaris in Figure 6d) were higher than the untreated membranes, but lower than the C_4_F_8_ plasma-treated wrinkle-patterned samples. In addition, the results also demonstrated that the combination of the high electron affinity fluorocarbon polymer and wrinkle pattern makes the fabrication process of the wrinkle pattern an effective and powerful method for the improvement of transparent TENG performance.

## Conclusions

In this work, we present a nanoscale fabrication process for wrinkle patterns and investigate its advantages systematically. The key feature of the nanoscale wrinkle-fabrication process is the use of a single-step fluorocarbon plasma treatment, which allows us to fabricate wrinkle patterns on different materials. By varying the plasma-treating time, we successfully obtained wrinkle patterns with a wavelength as small as 130 nm. After adjusting the plasma-treating time to control the wrinkle pattern, highly transparent wrinkle patterns were fabricated on PDMS and Solaris membranes, which proved the universality of the method. In addition, the deposition of fluorocarbon polymer effectively reduced the surface energy of the wrinkle pattern, making it applicable as a mold to more efficiently fabricate wrinkle patterns by replica molding. As a demonstration, we used it to form a highly transparent TENG with improved electric performance. This sing-step process to fabricate nanoscale wrinkle structures is universal applicable, low-cost, large-scale, controllable, and replicable, which leads to a strong potential for applications in flexible and transparent devices.
