# Supplementary material for: Controlled fabrication of nanoscale wrinkle structure by fluorocarbon plasma for highly transparent triboelectric nanogenerator
Source: Microsyst Nanoeng. 2017 Jan 30;3:16074. doi: 10.1038/micronano.2016.74 (PMC6444979; doi:10.1038/micronano.2016.74)
Supplement: Supplementary Information [file micronano201674-s1.pdf]

Supplementary file

Controlled fabrication of nanoscale wrinkle structure by fluorocarbon plasma for highly transparent triboelectric nanogenerator

Xiaoliang Cheng, Liming Miao, Zongming Su, Haotian Chen, Yu Song, Xuexian Chen and Haixia Zhang

Microsystems & Nanoengineering (2017) **3,** 16074; doi:10.1038/micronano.2016.74; Published online: 30 January 2017

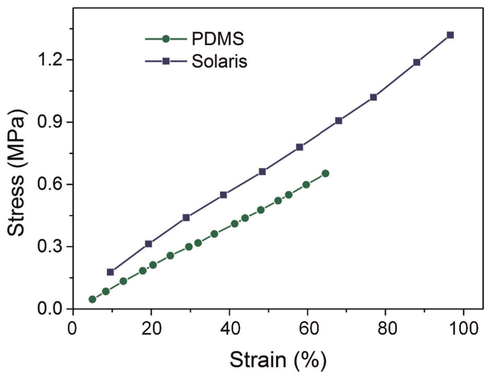

Figure S1 The stress-strain curves for PDMS and Solaris membranes. The Young's moduli of PDMS and Solaris membranes are measured using the stress-strain curves obtained by a pull and push dynamometer. Since the Young's modulus is the ratio of normal stress and normal strain, the moduli for PDMS and Solaris are caclulated to be 1.08 and 1.36 Mpa, respectively.

National Key Laboratory of Nano/Micro Fabrication Technology, Institute of Microelectronics, Peking University, Beijing 100871, China.
Correspondence: Haixia Zhang (zhang-alice@pku.edu.cn)
